# Text-Book of Diseases of the Kidneys and Urinary Organs

**Published:** 1896-03

**Authors:** 


### Reviews of Books.

**Text-book of Diseases of the Kidneys and Urinary Organs.** By Professor Dr. PAUL FÜRBRINGER. Translated by W. H. GILBERT, M.D. In Two Volumes. Vol. I. Pp. 194. London: H. K. Lewis. 1895.

The translation of this book will certainly meet a want, for it is not only a thoughtful treatise on diseases of the kidneys by a man of high abilities and great industry, but it supplies a readable account and criticism of the chief views held in Germany at the present time on renal matters. It is, perhaps, not a disadvantage that English and American authorities do not appear so often in these pages as Continental ones, since space is thus gained for the discussion of much valuable work too little known in this country. The translation is on the whole well done and clear, though here and there an obscure sentence appears, and German idioms are very frequent. It is a pity that care has not been taken to exclude such words and phrases as "typic nephritis," "cliniater," "it can cause in its generality," or again "these authors believe to have found the cause." Still, these blemishes are rare, and the meaning is, as a rule, well expressed.

The chief value of the book lies in the vast store of facts and observations from all quarters which the author has collected and discussed critically. A good example of this is shown in his review of the various theories of uræmia. The value of recent investigations is carefully pointed out; but he comes to the conclusion that uræmia is only a name for various disorders consequent on the retention of poisonous products, though what these substances are there is not as yet sufficient evidence to decide. Even then he remarks upon the difficulty that exists —though not perhaps an insuperable one—in accounting for uræmic disturbances which correspond to local cerebral dis-

[1] *Ibid.*, 166.

turbances. In the management of uræmia, he relies chiefly on diaphoretics, but mentions the failure that pilocarpin has been in his hands. Baths, careful purgation, and venesection have frequently proved of value.

After reviewing the work that has been done since his first edition in 1884, he classifies the forms of Bright's disease (besides circulatory disturbances) into acute and chronic diffused nephritis, contracted kidney, and amyloid degenerations. Thus he does not admit essential differences between parenchymatous and interstitial affections. The genuine contracted kidney, however, forms a distinct type produced by various processes, most often from a lingering nephritis. On the question of renal casts, he attaches importance to the presence of metamorphosed epithelial ones as showing decided disease; but he adds, rightly, that all casts are absent in many advanced cases. On the whole, we notice the great caution which leads him to reject as unproven many views which he acknowledges have much to be said in their favour.

We presume that with the second volume an index will be provided for the whole work.

